# 48th annual meeting and international conference of the Indian Society of Human Genetics 2024: fostering collaborations within rare disease research community

**DOI:** 10.1016/j.lansea.2024.100373

**Published:** 2024-02-23

**Authors:** Jayesh Sheth, Harsh Sheth, Frenny Sheth, Bittianda Kuttapa Thelma, Madhvi Joshi, Inderjeet Kaur, Chaitanya Joshi

**Affiliations:** a FRIGE's Institute of Human Genetics, Ahmedabad, Gujarat, India; b Department of Genetics, University of Delhi, Delhi, India; c Gujarat Biotechnology Research Centre, Gandhinagar, Gujarat, India; d L V Prasad Eye Institute, Hyderabad, Telangana, India

The 48th Annual meeting and international conference of the Indian Society of Human Genetics, jointly organised by FRIGE's Institute of Human Genetics and Gujarat Biotechnology Research Centre, was held from Jan 21 to 24, 2024, in Ahmedabad, India. This year's conference was special as it anthropomorphised the core tenets of human genetics: *Sarva mangalam bhavatu*, which means peace, health, and prosperity for all.

The conference was attended by over 700 delegates, including 130 faculties and 36 biotechnology companies from 13 countries who discussed and deliberated on the progress made in basic genetics, and translational aspects including screening, diagnosis, prevention, and novel treatment approaches in rare genetic disorders. With over 7000 such disorders, it is estimated that India may have ∼70 million cases most of which remain undiagnosed and for ∼95% of these, no approved treatment is available. These alarming statistics reflects burdensome lives of patients, families and healthcare fraternity who are deeply impacted by rare genetic disorders.

Presentations covering emerging genetic technologies like optical genome mapping, development of telomere-to-telomere reference genome and pan-genome analysis which have helped un-earth hidden complexities of the human genome and disease aetiologies were insightful.^1^ Evan Eichler sharing the latest understanding behind non-disjunction events through the use of Oxford Nanopore based methylation analysis; John Burn's exciting outcome of CAPP2 trials which demonstrated aspirin and resistant starch as cancer chemopreventive agents in Lynch syndrome^2^^,^^3^; Stylianos Antonarkis discussing the phenotypic consequences of causative variants, the penetrance of *FOXI3*-related craniofacial microsomia and the chromatin architecture of trisomy 21; Joris Veltman's exposition of two decades of data on the role of *de novo* variants in neurological disorders and male infertility^4^; and alarming data about global burden of birth defects and current approaches to their prevention presented by Kathleen Strong were the highlights of the plenary sessions.

Ongoing national and international focus on epidemiological studies understanding the incidence and distribution of genetic disorders such as inborn errors of metabolism, neurological disorders, hereditary cancer predisposition syndromes, neonatal screening programme and registry for rare genetic disorders by the Indian Council of Medical Research (ICMR) constituted thematic areas of discussion at the conference.

Whilst technological developments for genetic diagnostics have taken huge leaps, its applicability and utilisation has remained low in southeast Asia region. This is in part due to lack of awareness, screening and diagnostic protocols and lack of coverage of the costs by insurance companies or Government schemes associated with these tests. However, Department of Biotechnology (DBT), Government of India, has recently launched initiatives called NIDAN (National Inherited Diseases AdministratioN) and UMMID (Unique Methods of Management and treatment of Inherited Disorders), which are poised to offer genetic screening and diagnostics for common disorders through several centres and hospitals across rural areas of the country. More participation with public private partnership will make these programs more effective.

Along with improvement in diagnostic facilities, significant challenge in development and providing treatment for rare genetic disorders remain, especially in low-income and middle-income countries. High costs associated with treatments such as enzyme replacement therapies for lysosomal storage disorders and gene therapies for neuromuscular disorders, and many new emerging therapies make them less amenable for use in routine healthcare. To spur development in this sector, several national funding agencies such as the ICMR and DBT have taken steps to increase funding for indigenously developed drugs for rare genetic disorders. Additionally, repurposing of existing substrate reduction therapy and use of alternative supportive therapies need to be addressed as discussed by many eminent scientists.

Voices from all stakeholders of genetic healthcare: parents, caregivers, scientists, pharmaceutical industry, and State and Indian government were an invaluable experience and motivation to drive progress towards a singular goal of improving diagnosis of common and rare genetic disorders and developing indigenous low-cost treatments. This involved discussion of the National Rare Disease policy,^5^ establishment of centre of excellences for rare diseases (COEs) in all states, and new research and policy implementation programs for rare disease treatment, specifically enzyme replacement therapy for metabolic disorders and gene therapy for sickle cell disease, that are under development at both state and national levels in India.

The conference offered a wonderful platform for over 400 young researchers, 250 clinicians from all corners of India and abroad to display their latest scientific and technological advancements in understanding genetic disease aetiologies, disease biology and efforts towards development of novel therapeutics for several rare diseases; and facilitated interactions with the experts. The conference deliberations were focused on fostering cross-disciplinary and multinational collaborations within the rare disease research community in India. Let us hope more collaborations and a collective goal would help reduce the burden of genetic diseases and improve the health of all.

## Contributors

Conceptualisation of the idea: J.S., H.S., F.S., B.T., M.J., I.K., C.J.; Drafting the manuscript: J.S., H.S. and B.T.; Office bearers of the conference: J.S., H.S., F.S., B.T., M.J., I.K., C.J. All the authors reviewed and approved the final version of the manuscript.

## Declaration of interests

J.S. and H.S. were the conference chairman and organising secretary respectively and received the financial grants from Department of Science and Technology, Government of Gujarat; Science and Engineering Research Board (SERB) (SSY/2023/000766), Department of Biotechnology (DBT), Govt. of India (DBT/CTEP/01/20230754428); and Gujarat State Biotech Mission (GSBTM) (GSBTM/0002/01/2024 Dt: 01-01-2024), Council of Scientific and Industrial Research (CSIR) (SYM/12305/23-HRD), Indian National Science Academy (INSA) (SP/C-Dec-41/2023-24/) to conduct the conference. B.T. and I.K. received travel support from Indian Society of Human Genetics (ISHG) to attend the conference. H.S., F.S., B.T., M.J., I.K., C.J. were the office bearers of the conference and co-ordinated all the activities of the conference. The authors declare that the work was conducted in the absence of any commercial or financial relationships that could be construed as a potential conflict of interest.
